# Enhancing physicians’ radiology diagnostics of COVID-19’s effects on lung health by leveraging artificial intelligence

**DOI:** 10.3389/fbioe.2023.1010679

**Published:** 2023-04-20

**Authors:** Óscar Gasulla, Maria J. Ledesma-Carbayo, Luisa N. Borrell, Jordi Fortuny-Profitós, Ferran A. Mazaira-Font, Jose María Barbero Allende, David Alonso-Menchén, Josep García-Bennett, Belen Del Río-Carrrero, Hector Jofré-Grimaldo, Aleix Seguí, Jorge Monserrat, Miguel Teixidó-Román, Adrià Torrent, Miguel Ángel Ortega, Melchor Álvarez-Mon, Angel Asúnsolo

**Affiliations:** ^1^ Hospital Universitari de Bellvitge-Universitat de Barcelona, L´Hospitalet de Llobregat, Spain; ^2^ Department of Surgery, Medical and Social Sciences, Faculty of Medicine and Health Sciences, University of Alcalá, Alcala de Henares, Spain; ^3^ Biomedical Image Technologies, Universidad Politécnica de Madrid & CIBER BBN, ISCIII, Madrid, Spain; ^4^ Department of Epidemiology and Biostatistics, Graduate School of Public Health and Health Policy, University of New York, New York, NY, United States; ^5^ Campus Nord, Universitat Politècnica de Catalunya, Barcelona, Spain; ^6^ Departament d'Econometria, Estadística i Economia Aplicada-Universitat de Barcelona, Barcelona, Spain; ^7^ Department of Medicine and Medical Specialities, Faculty of Medicine and Health Sciences, University of Alcalá, Alcalá de Henares, Spain; ^8^ Ramón y Cajal Institute of Sanitary Research (IRYCIS), Madrid, Spain; ^9^ Service of Internal Medicine and Immune System Diseases-Rheumatology, University Hospital Príncipe de Asturias, (CIBEREHD), Alcalá de Henares, Spain

**Keywords:** radiology diagnostics, COVID-19, ICU, lung area, artificial intelligence

## Abstract

**Introduction:** This study aimed to develop an individualized artificial intelligence model to help radiologists assess the severity of COVID-19’s effects on patients’ lung health.

**Methods:** Data was collected from medical records of 1103 patients diagnosed with COVID-19 using RT- qPCR between March and June 2020, in Hospital Madrid-Group (HM-Group, Spain). By using Convolutional Neural Networks, we determine the effects of COVID-19 in terms of lung area, opacities, and pulmonary air density. We then combine these variables with age and sex in a regression model to assess the severity of these conditions with respect to fatality risk (death or ICU).

**Results:** Our model can predict high effect with an AUC of 0.736. Finally, we compare the performance of the model with respect to six physicians’ diagnosis, and test for improvements on physicians’ performance when using the prediction algorithm.

**Discussion:** We find that the algorithm outperforms physicians (39.5% less error), and thus, physicians can significantly benefit from the information provided by the algorithm by reducing error by almost 30%.

## 1 Introduction

During the last 3 decades, extraordinary achievements have been obtained in the field of Artificial Intelligence (AI). AI has been able to further improve its precision and the accuracy of its measures and predictions, due to the introduction of boosting models and the development of deep learning. Nowadays, AI has proven to be capable of performing tasks that previously were thought only humans could do (Fry, 2018).

Several disciplines have greatly benefited from these advancements (Grover et al., 2015; Kato et al., 2015; Zou, 2020). In these regards, the field of Medicine has not lagged behind. Many algorithms have been born in the recent years to help physicians improve their daily practice, mostly in the area of diagnosis for heart disease (Chen et al., 2020a), emphysema (Fischer et al., 2020), pulmonary nodules (Tandon et al., 2020), stroke (Lee et al., 2017), skin cancer (Kubota, 2017), breast cancer (Chen et al., 2010) or diabetic retinopathy in retinal fundus photographs (Gulshan et al., 2016).

A great change in healthcare needs has been observed in the last 2 years. The coronavirus (COVID-19) outbreak has imposed a great economic, political, social and medical challenge. The urgency to attend to an intense and sudden demand from COVID-19 has required a quick adaptation in diagnosis and treatment strategies, mostly in critical care intubated patients with oxygen therapy requirements. The saturation of human resources and medical materials requires innovation in the procedures and technologies used in patient management (Agazzi and Velázquez González, 2020; Mannucci et al., 2020).

In this paper, we propose to use convolutional neural networks, an innovative approach, to assess the severity of lung impact of COVID-19 patients by using exclusively chest radiographs and demographic patient data. Oxygen saturation is used to determine lung function, as it is the strongest known predictor of intensive care unit (ICU) use and death risk, with a relative weight of 20, 3%, once it is standardized by blood analysis data, comorbidities, age and sex/gender. Thus, we can infer how differentials in oxygen saturation correlate to lung health (Álvarez-Mon et al., 2021).

By using convolutional neural networks, the model identifies the lungs and extract three variables: pulmonary air density, pulmonary area, and presence of pulmonary opacities. These variables, selected to avoid a black-box algorithm and help medical interpretability, together with age and sex, are combined to assess the oxygen saturation level of the patient.

The main objective of the AI model is to determine the severity of lung affectation in COVID-19 patients. Our algorithm is parametric and fully interpretable, in contrast to other black box based approaches seen in the literature (Arun et al., 2020; Zhu et al., 2020; Ebrahimian et al., 2021; Mushtaq et al., 2021). Because of that we are able not only to achieve a high accuracy, but also to provide an estimated of the relative importance of each factor in the overall lung affectation.

Moreover, we undergo an experiment to find the best way of physician-algorithm cooperation, delving into a yet to expand field of how humans and algorithms can interact to attain better outcomes. Hence, we propose and evaluate two ways by which the model could help physicians to improve their diagnosis. First, when providing only the score of the algorithm. Second, when physicians get the score as well as the predictive variables used by the model. Finally, the results of these experiments are analysed.

The paper is organized as follows. First, we present the patients and methods used for the study. Then we present results of the predictive model, comparing them with respect to the results achieved by the human control group (composed by 4 radiologists and 2 internists) working alone and in the two trials that include different inputs of the model. Finally, we discuss our main results and conclude.

## 2 Patients and methods

### 2.1 Study design and patient’s cohort

The present study is designed as a two-stage study. The first stage corresponds to an observational, analytical, retrospective cohort study with longitudinal follow-up, to build an artificial intelligence-based algorithm to diagnose the level of oxygen saturation of a patient as a proxy of severity, based on radiographic images of the lungs. The second part aims to compare the performance of the model relative to a physician’s control group (composed by 4 radiologists and 2 internists), and to determine to what extend the combination of both (humans and artificial intelligence) could lead to better results. The conduct of the research and the dynamics of the study were carried out in accordance with the STARD Guidelines (Cohen et al., 2016).

The study population consists of retrospective consecutive recruitment of 1103 adult patients diagnosed with COVID-19 using polymerase chain reaction (PCR) test between March and June 2020. All patients were treated at HM Group, a group of seven hospitals in Spain. For each patient, available data consists of electronic medical records with age, sex, and at least one lung radiography taken within a 24-hours-time window before or after a measurement of basal oxygen saturation.

### 2.2 Modelling methods

#### 2.2.1 Image segmentation algorithm

To extract such information from radiographs, an algorithm involving image segmentation to identify lungs was designed. The algorithm is a four-step segmentation. First, a preliminary identification of lungs is performed using a lung detection deep learning algorithm. Second, given that the selected boundaries tend to contain irregular shapes and separated segments, the convex hull of the polygonal identification is computed. Third, a matching of an ideal mask representing the average lungs is performed on top of the previous prediction. Finally, a combination of the three previous segmentation is used as the final identification of the lungs. Next, we explain each of these steps in more detail.

Firstly, to identify the lungs from a given radiography, a Convolutional Neural Network (CNN) with U-net architecture has been trained consistent with Mineo and Mader (Mader, 2019; Mineo, 2019) using chest images from two datasets, the Montgomery County (Candemir et al., 2013) and the Shenzhen Hospital (Mineo, 2019)These datasets have as a target lung segmentation. Previous segmentation methods such as the work of Novikov et al. (2017) (Novikov et al., 2017) train a CNN to also segment the heart to detect better the lungs. However, since our training datasets did not have heart segmentation data, we segmented only lungs and refine the predictions with the convex hull and the ideal mask transformation. Our training strategy relies on the strong use of data augmentation to improve the efficiency of the available annotated samples from both datasets. All chest radiographies are pre-processed inverting the radiographies depending on their photometric interpretation, resized to 512x512 pixels and scaled to a 0–1 scale (i.e. 1 the brightest pixel and 0 the darkest in order to standardize the image intensity in each of the images). Data augmentation and pre-processing has been created based consistent with previous work (Mineo, 2019; Candemir et al., 2013). The CNN achieved over 90% binary accuracy (defined as whether two pixels in the image match or not) on both the training and validation data of both datasets.

Secondly, the convex hull (that it is, the smallest convex polygon enclosing all the points of a given shape) of each of the two principal components of the previous segmentation are selected. The convex hull is performed using the OpenCV implementation of Sklansky’s algorithm (Sklansky, 1982).

Thirdly, on top of the net prediction, it is found the best affine transformation *A* that minimizes a similarity measure, the enhanced correlation coefficient criterion between the standard morphology of a lung selected from a very clear chest radiography and segmented in detail by a group of physicians and the net segmentation following the optimization algorithm proposed by Evangelidis (Evangelidis and Psarakis, 2008) as implemented in OpenCV (Bradski and Kaehler, 2000). Since the sample of patients consists of adult people (mean 66 ages, standard deviation 15 years, and a minimum of 20 years), there is no need for a pediatric ideal lung segmentation. Then, the standard morphology is transformed according to *A* obtaining a third lung segmentation. As a robustness check, this step was validated by quantifying the amount of rotation in terms of the L2-norm of the rotation matrix. Manual inspection of the distribution of this value found that most of data lied in the 0–1 interval. In addition, values greater than 2 were found to be outliers with an incorrect lung identification and were dropped.

**FIGURE 1:**
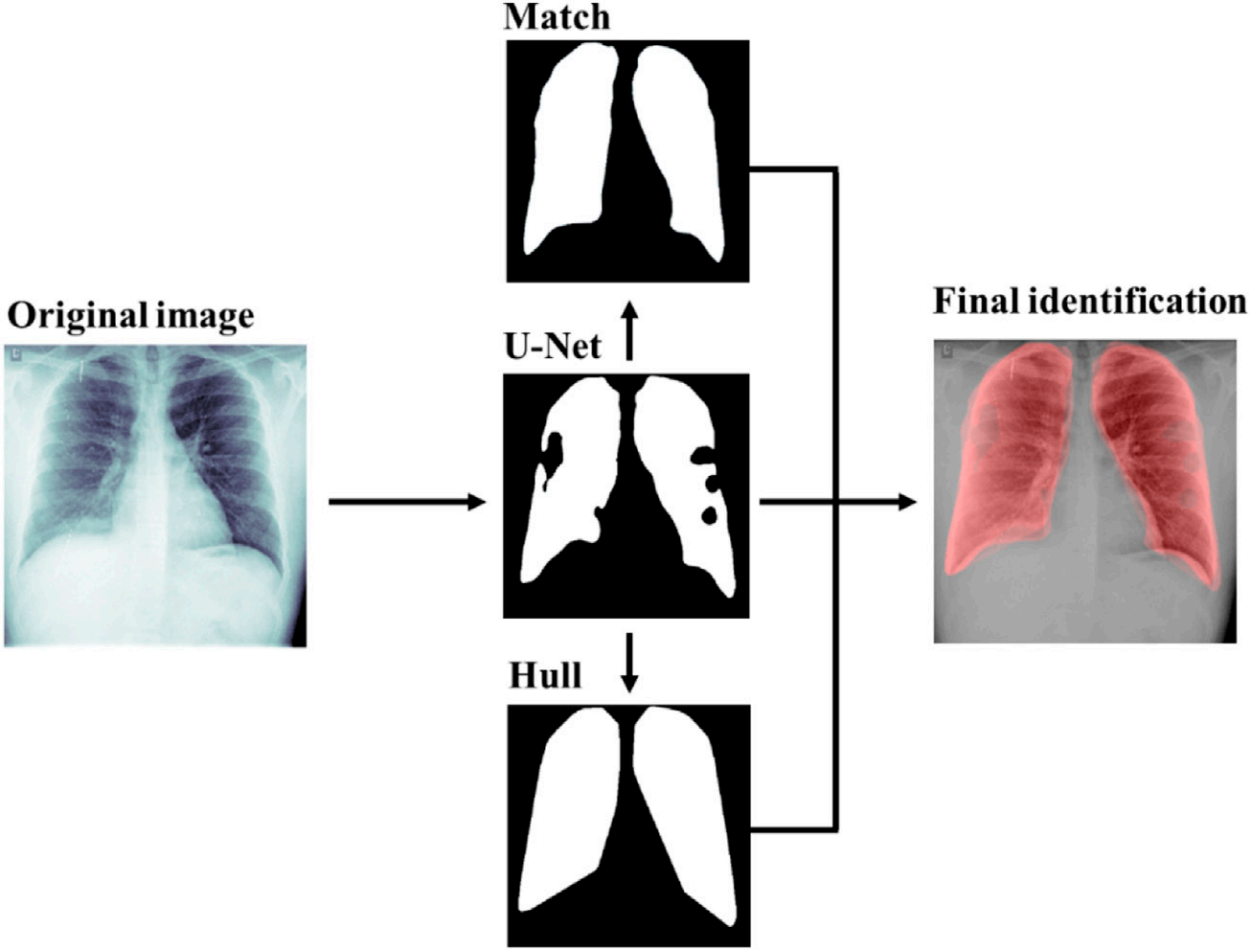
Lung segmentation of a 21-year-old male with adequate saturation level. Segmentation steps include the U-Net prediction, the standard lung matching and the convex hull of the prediction. Results are combined into the final identification.

The final prediction of our algorithm is the weighted average of the three previous steps. Optimal weights were found by the maximization of the Jaccard index in a 10% resolution grid with respect to the manual labelled data in the Montgomery County dataset (Álvarez-Mon et al., 2021). These weights are 30% standard morphology, 60% U-Net prediction and 10% convex hull. Figure 1 shows an example of the different steps and its outputs of our lung identification algorithm. The example shows how the CNN-U-Net prediction improved by the convex hull and the transformed standard lungs (Match). Empirical results show a decrease of a 10% of relative error on the labelled data from Montgomery County Dataset (Candemir et al., 2013). Moreover, the convex hull and the standard morphology helps the physicians, in the sense that the output lung segmentation has a full lung shape instead of a shape with holes on it. Details of the estimation of the sample size needed to achieve a certain level of signification are provided in the appendix. In particular, see Supplementary Table SA for this case where the improvement has approximately an 80% significance (Supplementary Appendix SA).

#### 2.2.2 Oxygen saturation predictive model

Here we describe the variables used to build a predictive model of the oxygen saturation of the patients, based on their radiographic images of the lungs.

##### 2.2.2.1 Variables

For each patient, we have medical records including age, sex, and results of basal oxygen saturation level at an interval of 24 h before or after the radiology were taken.

##### 2.2.2.2 Target

In the context of the first coronavirus outbreak (from March to May 2020), four-level categories were established for the target of our model, the oxygen saturation, as predictors of ICU admission or death, based on our previous work (Álvarez-Mon et al., 2021).

1. Extremely low level corresponds to less than or equal to 80%.
2. Low level between 81% and 90%.
3. Medium between 91% and 94%.
4. Adequate to >94%.

The model target is then calculated as a transformation of the four categories into a numeric value standing for severity. The severity is constructed using the coefficients of the EM-8 model from a previous study using the same data to predict the risk of ICU admission or death (Álvarez-Mon et al., 2021). Specifically, coefficients 2.4006, 1.0468, 0.6528 and 0.0000 were assigned to extremely low, low, medium, and adequate saturation, respectively. Assigning the same target value to each patient of a group helps minimizing the variance of the model error and better identifying different groups. Moreover, having a high value for the extremely low patients helps the model by improving the distinction of highly affected patients.

Pneumonia-affected lungs or low saturation lungs tend to present visual signal in terms of overall brightness, lower transparency areas and presence of brighter spots or opacities (Cura, 2011). Following this evidence, three variables were computed from the lung segmentation analysis, which are the *pulmonary air density*, the *lung area* and the *opacity area*. These variables were also interesting to use as the model has medical interpretability, avoiding making it a black box for physicians.

#### 2.2.3 Pulmonary air density

**FIGURE 2:**
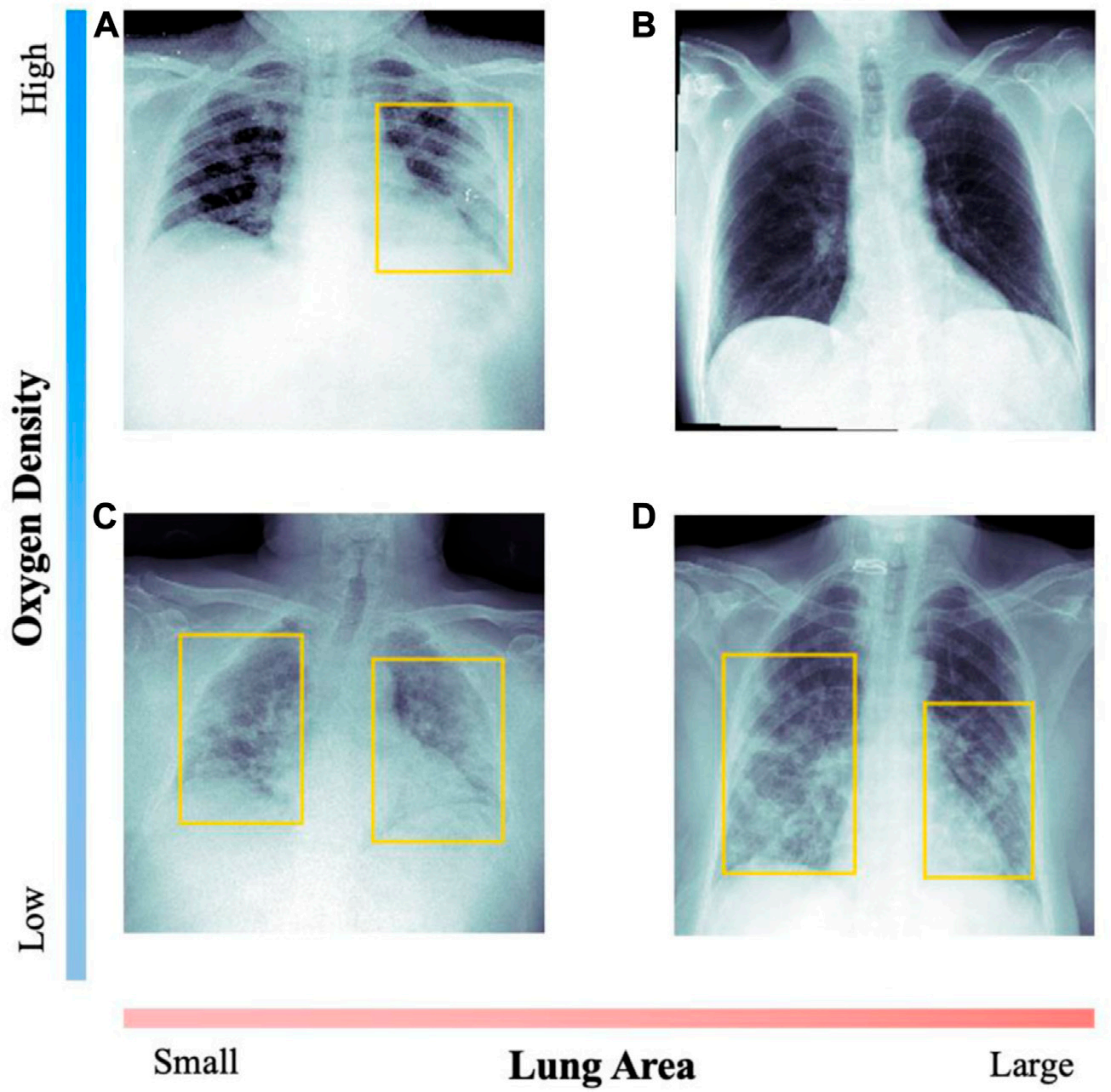
Comparison of radiographs with opposed values on the diagnosis variables. For each image, the patient’s data is included. Yellow boxes indicate lung opacity detection. Image **(A)** corresponds to a 47-year-old woman, with medium saturation; **(B)** to a 53-year-old man with adequate saturation; **(C)** to an 82-year-old man with extreme saturation; **(D)** to a 72-year-old woman with low saturation.

The pulmonary air density corresponds to the global level of air density observable in the radiography. We estimate this value as the difference of the median pixel intensity of the body and the median intensity of the identified lungs. That is, computing the median pixel intensity in the 0–1 scale of the body and the identified lungs, and then performing the difference of such two values. For the difference variable, the values are in the −0.6–0.5 range with an average value of 0.14 and standard deviation of 0.06. For healthier patients, this value is expected to be positive, as the lungs appear darker than the rest of the body in most radiographies. Figure 2 shows two examples of images with opposite densities. Values are standardized with respect to the average density of a healthy patient.

##### 2.2.3.1 Lung area

Lung area corresponds to the ratio of lung area with respect to the total image, as detected by the lung segmentation algorithm. While this value could be partially incorrect due to different zoom in the images, it constitutes a good approximation of the lung area as we show in the results section. The values are in the 21.1%–50.2% range with an average value of 33.1% and standard deviation of 7.2%. Figure 2 shows two examples with very different lung area.

##### 2.2.3.2 Opacities

Opacities are brighter spots in the image that could correspond to visual signal of pneumonia affectation. To detect lung opacities, a Mask Region-based CNN designed by He et al. (He et al., 2017) was trained to detect visual signal of pneumonia in medical images on the dataset provided by the Radiological Society of North America (RSNA) (Mader, 2019; Mineo, 2019; Mader, 2019; Mineo, 2019). The dataset contains 30,000 images. In 6,012 of those images, 9,555 lung opacities were identified and labelled, with their corresponding bounding boxes. Training was performed following the open-source work of H. Mendonça (2019) (Rsna, 2019) using the pre-trained weights with the COCO dataset (Lin et al., 2014). All the hyperparameters and the training strategy were replicated from his previous work, resizing the chest radiographs to 256x256 pixels and using the same data augmentation as a training strategy. The Mask placed top 10% on the RSNA 2018 Challenge R-CNN, and achieved a 20% mean average precision, slightly below the best model (25% mean average precision) (G. Shih et al., 2019) (Shih et al., 2019).

In Figure 2 we compare radiographic images with clear visual signal of opacities and healthier examples, with undetectable affectation.

##### 2.2.2.3 Model and interpretation

The model consists of a multivariable linear regression estimated through Ordinary Least Squares (OLS). The target is the numerical value of the oxygen saturation levels, and the covariates were age, sex, pulmonary air density, opacities and lung area. The model was trained using 1578 radiographies from 892 patients, selected from a group of hospitals within HM, and tested with 374 radiographies from 211 patients from another group of hospitals. The train and test datasets were selected randomly using an 80%–20% split.

To interpret the model beyond the significance of the parameters, we estimate the relative importance of each variable included in the model, by modelling the oxygen saturation level in a linear regression including the covariates stated in 2.2.2.1. and using SHAP (SHapley Additive ExPlanation) values (Lundberg and Lee, 2017; Lundberg et al., 2018).

### 2.3 Experiment design for human-AI collaboration to assess the diagnosis predictive values

The experiment was performed in three parts. Each of them was conducted on a set of 100 radiographs formed by 50 pairs of radiographs of the same patient to evaluate both, oxygen saturation (Extremely low, Low, Medium, or Adequate) and evolution (Worsen, Stable, Improve) of the COVID-19 pneumonia affectation. Six physicians evaluated the saturation level of the first 50 radiographs of each patient, and then they evaluated the remaining 50 radiographs and patient’s evolution, based on both radiographs, without sharing their answers with each other, allowing us to check their diagnostic agreement.

Finally, a total of 900 radiographs from 450 patients were evaluated by only two of the six physicians. Each physician evaluated 300 radiographs from 150 patients for a total of 1800 evaluations. To avoid bias, the radiographs used for testing the experiments had not been used previously neither for training the model nor seen by any of the six physicians.

Experiment 1: Comparison of algorithm *versus* physicians without assistance.

For the first sample of 100 radiographs, physicians only had patients’ lungs radiograph and information about their age and sex, without any guidance from the model output.

Experiment 2: Comparison of algorithm *versus* physicians with assistance of the algorithm’s final score.

For a different sample of 100 radiographs, physicians had to diagnose the saturation level as in the previous experiment, but they also had model’s predicted saturation level to guide their decision for the final diagnostic.

Experiment 3: Comparison of algorithm *versus* physicians with assistance of the algorithm’s final score and the diagnostic variables.

The last experiment consisted of a new sample of 100 radiographs, where physicians had the same information as in the Experiment 2 and other relevant information: a picture overlapping patient’s radiograph identifying lungs’ area, a picture overlapping the radiograph identifying lungs’ opacities, and a summary table with patients’ percentage of opacities, lungs’ area and predicted saturation compared with the mean values of healthy people of the same gender and age.

To assess the impact of the algorithm on physicians’ performance depending on the level of assistance, we calculated the Root Mean Square Error (RMSE) and the Area under the Curve (AUC; considering the target as whether the patient has low or extreme saturation) of the assessments made by the physicians in the different experiments and compared with those of the algorithm. For unbiased comparison, the algorithm was neither trained with the radiographs of the experiment, nor retrained with physician’s answers. Moreover, since physicians were asked to label the level of oxygen saturation in four groups, the prediction of the model was also categorized by assigning a threshold to each of the four categories of saturation, keeping the original proportion of each label in the training set and the predictions. That is, threshold values were selected to match the same proportions in the predictions as in the original classes. Namely, we selected the severity to be < 0.6 adequate, 0.6–0.8 medium, 0.8-1 low and >1 extremely low.

### 2.4 Ethics and approval

This study was conducted according to basic principles of ethics (autonomy, harmless, benefit, and distributive justice). The protocol was in line with the standards of Good Clinical Practice and the principles of the last Declaration of Helsinki (2013) and the Oviedo Convention (1997). Ethics committee approval was obtained from the University Hospital Príncipe de Asturias (HUPA-04062020).

## 3 Results

### 3.1 Development of a diagnostic algorithm for the assessment of pulmonary involvement in COVID-19 pneumonia

We began our study by investigating the incidence of alterations in the three elements of our model measurement in 1103 patients diagnosed with COVID-19. The COVID-19 patients were stratified according to their pulmonary functional severity as measured by the oximetry they presented at the time of radiography (Table 1). Specifically, 575 patients have only one radiography, while 528 patients have at least two radiographies; for a total of 1952 radiographies.

**TABLE 1:** Description of the characteristics (%) for COVID-19 patients included in the study.

	Total patients (n = 1103)
Age (SD)	66.0 (15.6)
Female	38.2
Oxygen saturation extremely low	2.6
Oxygen saturation low	22.4
Oxygen saturation medium	43.0
Oxygen saturation correct	32.0

Table 2 shows the average value of the different values according to the saturation levels of the different patients. For better interpretability, data is standardized with respect to the average healthy patient having adequate level saturation. The percentages represent the existence of alterations in the radiographs of patients in advanced stages of pneumonia with respect to those with normal saturation. The mean age and the percentage of female gender in each of the stages of COVID-19 pneumonia are included.

**TABLE 2:** Average values (%) of the study variables per saturation level. Gender is expressed as the percentage of women. For better interpretation, image diagnostic variables are expressed in terms of deviation from the average healthy patient (hence, the 0% values in the adequate level).

	Saturation (%)	Age	Sex (%)	Pulmonary air density	Lung area	Opacity (%)
Adequate	96.0	59.6	52.1	0.0%	0.0%	0.0
Medium	92.7	64.9	32.1	−2.7%	−3.6%	18.8
Low	87.3	69.0	30.9	−13.2%	−5.9%	72.2
Extreme	77.8	78.3	25.0	−19.8%	−15.4%	89.9

As shown in Table 2, it was observed that the detection of alterations in the three variables analyzed in the model increased with the severity of the functional repercussion of the patient’s pneumonia. As expected, the lower the oxygen saturation, the lower the density, the lower the lung area, and the higher the opacities. Of note it was the marked increase in pulmonary opacity, reaching 89% in the most severe forms.

To evaluate the severity of COVID-19 pneumonia on patients based on chest-radiographs, we built a regression model to analyse the importance of the five variables, which included the patient’s age and sex, the surface area, density and opacity of the lung radiograph with respect to the saturation estimate presented by the patient. Older age, male, smaller the lung area and greater the surface area with lung opacity were associated with lower oxygen saturation. However, lung density was not significant. We also calculated the predictive value, measured as AUC of the model, on the diagnosis of saturation states below 81% and 91% obtaining a score of 0.792 and 0.736, respectively on the 1578 images of the training dataset. The RMSE was 0.505 (Table 3). On the test dataset, consisting of 374 images, the results have been found to be consistent, with an 0.779 and 0.724 AUC and a 0.603 RMSE.

**TABLE 3:** Relationship of the model variables with oximetry. In a total of 1578 radiographs, the association of these variables was analyzed by means of multiple linear regression with the figures obtained in pulse oximetry. Estimated parameters of the models. Standard errors in brackets. AUC = area under the curve, where the target is “Extreme” or “Low” saturation.

	Estimates	*p*-value
Age	0.0081 (0.0008)	<0.0001
Sex	−0.1329 (0.0283)	<0.0001
Lung area	−1.2367 (0.1858)	<0.0001
Pulmonary air density	−0.3690 (0.2188)	0.0919
Opacity area	0.8272 (0.1446)	<0.0001
Train: Number of observations	1578	-
Train: RMSE	0.505	-
Train: AUC (Extreme or Low)	0.736	-
Test: Number of observations	374	-
Test: RMSE	0.603	-
Test: AUC (Extreme or Low)	0.724	-

It is important to highlight that the average inflation factor (VIF) of the covariates was 1.20 and the maximum was 1.33 suggesting that there was no evidence of multicollinearity.

Finally, to gain a complete understanding of the model beyond the significance of the parameters, we estimated the relative weight of each variable included in the model using SHAP values Equation 1. Table 4 represents the relative importance of each variable in the diagnostic severity model. The highest relative importance is for Age (31.8%), followed by opacity area (20.6%) and Lung area (22.8%). However, smaller values of sex (16.0%) and pulmonary air density (5.8%) have relative low importance.

**TABLE 4:** Relative importance (%) of the variables in the total cohort of COVID-19 patients based on SHAP values.

Age	31.8
Lung area	22.8
Opacity area	20.6
Gender	16.0
Pulmonary air density	5.8

### 3.2 Diagnosis enhancement experiment

**FIGURE 3:**
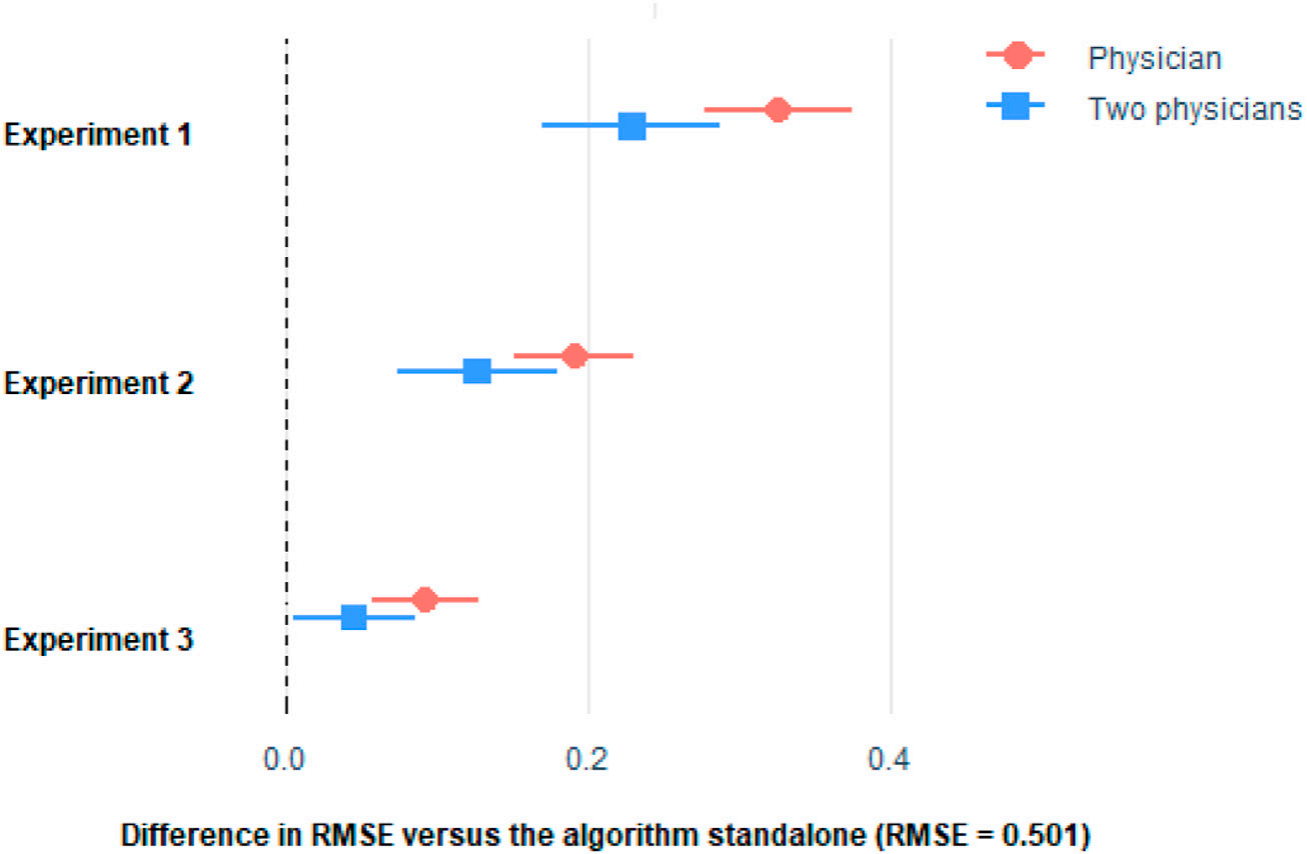
Difference in RMSE with respect to the algorithm of the stratification made by the physicians only, and the mean of two physicians. Horizontal lines represent the 95% CI of the difference. See Supplementary Table SA of the Appendix for the procedure to build the 95% CI.

**FIGURE 4:**
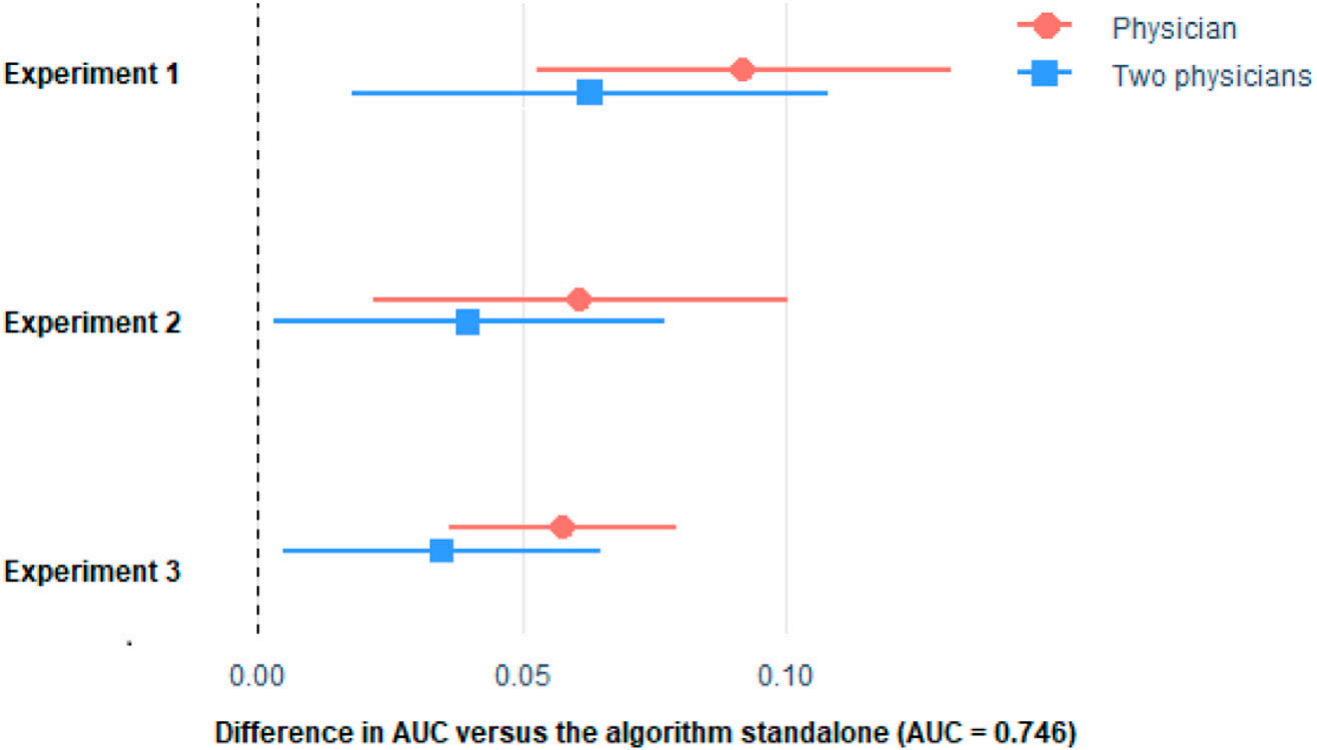
Difference in AUC with respect to the algorithm of the stratification made by the physicians only, and the mean of two physicians. Horizontal lines represent the 95% CI of the difference. See Supplementary Table SB of the Appendix for the procedure to build the 95% CI. Please see comment on previous figure regarding footnote.

We investigated the potential diagnostic value as a unique or radiologist support tool of our algorithm in the severity assessment of radiological imaging of COVID-19 pneumonia. First, we analyzed a total of 300 radiographs each interpreted by two different physicians. For this assessment, physicians only had patients’ lungs radiograph and information about their age and sex. Figures 3, 4 show that the predictive ability of the algorithm to predict patient severity was significantly higher than that of the individual physicians and even higher than the mean of those performed by each pair. Comparison of algorithm *versus* physicians without assistance.

We then investigated the impact of knowledge of the application of the diagnostic algorithm on the radiologists’ assessment of severity. In 300 new radiographs examined by pairs of physicians, we found that the predictive ability of the patient’s oximetry was significantly lower both in the individual physician analysis and when acting in pairs than that achieved by the algorithm. However, there was a marked improvement in the radiologists’ assessment. In assessment test 3, a similar approach to test 2 was followed, with another 300 new radiographs, but the physicians had both the numerical data and their graphical representation. The results show that significant differences persist in the predictive ability of the physicians with respect to the algorithm, but the differences are attenuated and improved with respect to the previous assessments.

As it can be seen, the algorithm outperformed the physicians in the three experiments. Remarkably, physician’s performance improved with the more the information they get from the algorithm, but they were not able to surpass the algorithm. Even if we considered the average of the predictions made by two physicians (Figures 3, 4), the combined score cannot outperform the algorithm alone.

### 3.3 Experiment to improve the diagnosis of clinical evolution

We calculated the percentage of cases where the physician evaluation of a pair of radiographs of the same patient at different time, when presented individually, was not consistent with the evaluation of patient’s evolution. For instance, this would be the case when the physician initially diagnosed as “Low” a radiograph of a patient, then 2 days later labelled as “Extreme” a radiograph for the same patient, but when asked for the evolution of the patient comparing the two images, answered that the patient had a “Positive” evolution.

The inconsistency of intra-physician rating was considered whether either the physician stated that the patient was “Stable” in terms of evolution (when having the two radiographs at the same time) but labelled the radiographs differently when assess them separately (that is, for instance, labelled the first one as “Medium” and the second as “Extreme”), or the physician stated that the patient had a “Positive” or “Negative” evolution, but labelled the radiographs as it was the opposite. In another words, the first radiograph received a better label than the second one when evaluated separately for “Positive” the evolution, or the other way around in case she stated a “Negative” evolution. Due to the model’s assistance, there is a 55.4% and 61.1% reduction of inconsistences due to the assistance of the model (Table 5).

**TABLE 5:** Intra-physician, Inter-physician and Physicians compared with algorithm percentage of inconsistences (I, %) and relevant inconsistences (RI, %) in each experiment. For experiments 2 and 3 also expressed relative reduction of inconsistences with respect to experiment 1 expressed in column “% diff”. For intra-physician, the percentage of inconsistences is estimated over the 50 sets of two radiographs of the same patient (150 sets in each experiment), while for the inter-physician is over the 100 radiographs (300 in each experiment).

	Intra-physician		Inter-physician			
	I (%)	% diff	I (%)	% diff	RI (%)	% diff
Experiment 1	6.0	-	60.3	-	13.3	-
Experiment 2	2.7	−55.4%	44.7	−25.9%	3.0	−77.4%
Experiment 3	2.3	−61.1%	41.3	−31.5%	1.0	−92.5%

Regarding inter-physician inconsistencies, we calculated the percentage of cases where the two physicians who evaluated the same radiograph in the same experiment were not consistent (i.e., their evaluations of the saturation level were different), especially those cases with relevant inconsistences (i.e., two or more saturation levels of difference). The results show a 25.9% and 31.5% of inconsistences’ reduction, and 77.4% and 92.5% for relevant inconsistences (Table 5).

## 4 Discussion

In this paper, first we develop an individualized artificial intelligence model to help radiologists assess the severity of COVID-19’s effects on patients’ lung health, including prediction for fatality (death or ICU admission). Then we use the developed algorithm to test different ways of physician-algorithm cooperation, finding that, in this specific case, the algorithm outperforms physicians (39.5% less error), and thus, physicians can significantly benefit from the information provided by the algorithm by reducing error by almost 30%.

In healthcare practice, the interpretation of radiological images is subject to variations among the professionals who perform them (Onder et al., 2021). Consistent with previous studies regarding radiologic bias and human error (Singh et al., 2013), we found a discrepancy in inter-observer and intra-observer rating severity assessment of radiological imaging of COVID-19 pneumonia among physicians of 60% and 6%, respectively. In the case of discrepancies between two physicians, there was a 13% discrepancy rate involving two or more levels on the severity scale of COVID-19 pneumonia, which could resolve in a change of the patient’s management. These findings were similar to the discrepancies found in previous studies (Buchanan et al., 2004; FitzGerald, 2005; Al-Khawari et al., 2010; Cohen et al., 2020). In this regard, when assisted by the algorithm, we found a large reduction in inter and intra-physician discrepancies, meaning that the model assistance tends to unify physicians’ decision on COVID-19 pneumonia patients’ diagnostic.

It is important to highlight that, besides achieving a higher goodness of fit than physicians, the algorithm is also consistent, in the sense that it always evaluates the images the same way. In contrast, physicians are not that consistent, either with respect to themselves in previous time (intra-physician inconsistencies) or with respect to their colleagues (inter-physician inconsistencies), as previously indicated.

Many previous studies compare the AI radiologic findings with the Radiographic Assessment of Lung Edema (RALE) score, which was designed to measure the extension and severity of acute respiratory distress syndrome (ARDS) (Lundberg et al., 2018). In our multicentre experience, this scale is rarely used in day-to-day hospital practice, hence, we chose to let the physicians in our study to determine the stage of severity based on their own professional experience. This fact led to an increased variability in the human radiologic report of results, but also correlates better with the hospital reality.

In this work to identify the lungs from a given radiography, a CNN with U-net architecture has been trained (Mineo, 2019) (Mader, 2019) using chest images from two available datasets (Mineo, 2019) (Candemir et al., 2013). These datasets have as a target lung segmentation. Thus, we performed a novel analysis method that segmented only lungs and refine the predictions with the convex hull and the ideal mask transformation (Arun et al., 2020; Chen et al., 2020b; HuYuan and Zhangming, 2020; Wu et al., 2020; Zheng et al., 2020; Zhu et al., 2020; Ebrahimian et al., 2021; Mushtaq et al., 2021; Roberts et al., 2021; Chao, 2023). Furthermore, despite relying on deep learning for the segmentation of the lungs, in order to increase the amount of trust a physician could have on the predicted COVID lung disease prognosis, we decided to not rely on a complete black box system. Thus, we decided to include only features from the radiographs that can be understood from the medical perspective and use them later as features for an interpretable model. Other studies using AI to enhance chest radiograph COVID-19 diagnostics are black box based (Arun et al., 2020; Zhu et al., 2020; Ebrahimian et al., 2021; Mushtaq et al., 2021; Yang et al., 2022). Other published papers extract handcrafted radiomic features (Chen et al., 2020b; HuYuan and Zhangming, 2020; Wu et al., 2020; Zheng et al., 2020). All these papers aimed to predict patient prognosis, using endpoints such as death, ICU admission, need for ventilation, or progression to acute respiratory syndrome. None of the aforementioned papers took the interpretability and understandability of the AI models as a main objective. Conversely, at the opposite end of the spectrum, in contraposition of the black box based AI, a new research topic is emerging, known as explainable AI (XAI) (Ye et al., 2021; Wu et al., 2020). In high responsibility fields such as medicine, the need of understandable AI models is crucial for decision-making. Following this emerging trend, the development of a parametric AI such as the one designed in this study, not only considers the overall comprehensiveness of the algorithm by its user, but the possibility to learn from the AI become readily available for the physician using it. For instance, as a result of this approach, we are able to know that demographic patient data (age, with a 31,8% of relative weight, and sex, with a relative weight of 16%) accounts for 47.8% of the weight in predicting fatality outcomes, and radiographic variables for the remaining 52.2%. When focusing on radiographic data, lung area becomes the most important predictor, with a 22, 8% of the relative weight, followed by the total area of opacities, with 20, 6%. Those two variables are extensively more important than pulmonary air density, which accounts for 5,8% of the relative weights. The capacity to measure the importance of a radiologic finding for the patient’s outcome conforms a very useful tool for the radiologist and the multidisciplinary patient’s management.

This suggests that the design of algorithms using clear parametric data to achieve its results are better trusted by physicians, and can help to learn, train and improve physician’s performance. Unfortunately, in this study we did not quantify the improvement in performance of physicians without assistance before and after having interacted with the algorithm.

Amongst the limitations of this study, it is important to remark that this AI is not intended to replace the role of radiologists or the serialized oxygen saturation tests. It was created as a complement to aim in the management decision, as a tool to extract parametric radiologic knowledge, and as an instrument to test better ways to enhance human-AI interactions. Second, we did not test the accuracy of the algorithm in non-COVID patients. Further research may verify its utility in the assessment of other pathological contexts of lung affectation. Third, we did not compare the performance of our algorithm *versus* other algorithms. Another limitation arises when we try to compare our results with other papers with similar objectives. For instance, in our study, using demographic data, SpO2 and chest radiographs we reach an AUC of 0.736. In another study (Wu et al., 2020) with similar objectives as ours (prediction of fatality as death or ICU admission), they found an AUC of 0.884, using CT scan, wider arrange of laboratory data and demographic data. Another comparable study (Xie, 2020) in terms of defined objectives of algorithm prediction, found an AUC of 0.862 using only CT scan data. Yet another study found in the literature (Driggs, 2021), with similar endpoints, reached an AUC of 0.880, based on CT scan, demographic and other laboratory data. Any attempt to make a reliable comparison between these studies becomes very difficult due to the use of different population samples, different protocols of ICU admission and different variables used in prediction model construction. As a side note, despite the fact taht our model achieves a lower AUC than the previously mentioned studies, we’d like to stress that our variables (age, sex, chest radiograph and SpO2) are quicker to obtain and more cost-efficient in an emergency room admission context, finding a similar AUC than those other studies.

Finally, another limitation we should take into account is that our database includes exclusively patients of the first COVID-19 wave, which consisted mainly of elderly people. In the following COVID waves other age groups were also affected. For this reason, our results are mainly focused on older people. Characteristics of younger groups may not be addressed in our algorithm.

In front of the many AI and COVID related papers that have been published in the recent years, concerns about development of AI in medical imaging have been raised and renewed (Chao, 2023). The need for checklists that guarantee the quality and reproducibility of such AI models is of the utmost importance. This study aspired to meet those requirements: robustness checks were made, demographics of our data partitions was reported, as well as the statistical tests used to assess significance of results, and a multidisciplinary team of physicians, physicist and mathematicians was ensembled for this project.

## 5 Conclusion

Additionally, it is important to note that the assessment of COVID-19 severity based exclusively on radiographs is not performed in the day-to-day hospital practice, and thus, further research should be conducted to show if cooperation between radiologists and algorithms in rutinary image diagnostics can outperform the results of either one alone.

Finally, we can conclude that the use of models, visual and numerical, generated by AI have been shown to be useful both in improving the diagnosis of pneumonia severity and in decreasing variability, becoming a useful aid for clinicians.

## Data Availability

The data analyzed in this study is subject to the following licenses/restrictions: Not applicable. Requests to access these datasets should be directed to angel.asunsolo@uah.es.
