# Supplementary material for: Enhancing physicians’ radiology diagnostics of COVID-19’s effects on lung health by leveraging artificial intelligence
Source: Front Bioeng Biotechnol. 2023 Apr 20;11:1010679. doi: 10.3389/fbioe.2023.1010679 (PMC10157246; doi:10.3389/fbioe.2023.1010679)
Supplement: Supplementary file 1 [file Table1.docx]

**Appendix**

***Significance of the performance difference between agents***

In a small sample set, a worse-scoring agent might obtain a higher value in a metric than a better agent with some probability. Also, the inverse phenomena may happen, a better-scoring agent might obtain a lower value in a small sample size but be better on the big dataset. This is due to the randomness inherent to the prediction process. We designed an algorithm to compute the probability distribution of a worse agent to score higher than a better one, depending on the sample size, and the how much worse is the agent.

Over the ground truth, we generate alternative predictions by introducing Gaussian error to the predictions made by the predictive model (PM). Hence, the agents should be worse than the PM. We then create random samples of size $n_{j},$ much smaller than the total data size. An agent with correlation $\rho_{i},$ Root Mean Square Error (RMSE) $r_{i}$ and Area Under the Curve (AUC) $a_{i}$ respect to the target “Extreme” or “Low” saturation over the total population will have a probability $p_{ij}$ to score lower than the PM in the original dataset. We compute $p_{ij}$ by taking samples of size $n_{j}$ and simulating agents with correlation $\rho_{i}$, $r_{i}$ and $a_{i}$ repeatedly and calculating the average of times that the PM beats the agent on the original dataset. Each one of the probabilities have been averaged over more than 5000 simulations in order to obtain enough simulations for the 0.001 p-value. This led us to perform more than 1 million agent simulations.

The values were computed for different agents having growing differences in correlation and RMSE with respect to the best model. Table A shows the result for different sample sizes and correlations, each cell representing the probability $p_{ij}$. Table B shows the results for different sample sizes and RMSE differences and Table C shows the results for different sample sizes and AUC differences.

**Table A.** Probability that the PM scores higher than worse agents on the original dataset. The different rows represent differences in points of correlation with respect to the best model over the sample size dataset. Sample sizes, representing each column, range from 30 to 150.

|  | **30** | **40** | **50** | **60** | **70** | **80** | **90** | **100** | **120** | **150** |
| --- | --- | --- | --- | --- | --- | --- | --- | --- | --- | --- |
| **1%** | 0.675 | 0.675 | 0.673 | 0.692 | 0.713 | 0.727 | 0.710 | 0.719 | 0.757 | 0.790 |
| **2%** | 0.747 | 0.736 | 0.783 | 0.793 | 0.809 | 0.843 | 0.831 | 0.850 | 0.854 | 0.878 |
| **3%** | 0.743 | 0.741 | 0.753 | 0.789 | 0.811 | 0.829 | 0.862 | 0.864 | 0.876 | 0.908 |
| **4%** | 0.746 | 0.791 | 0.820 | 0.849 | 0.870 | 0.874 | 0.862 | 0.903 | 0.914 | 0.933 |
| **5%** | 0.805 | 0.836 | 0.868 | 0.876 | 0.888 | 0.918 | 0.928 | 0.937 | 0.939 | 0.965 |
| **6%** | 0.763 | 0.801 | 0.823 | 0.834 | 0.871 | 0.904 | 0.928 | 0.903 | 0.930 | 0.953 |
| **7%** | 0.829 | 0.856 | 0.875 | 0.894 | 0.908 | 0.935 | 0.942 | 0.939 | 0.970 | 0.984 |
| **8%** | 0.833 | 0.871 | 0.907 | 0.915 | 0.920 | 0.956 | 0.946 | 0.970 | 0.978 | 0.983 |
| **9%** | 0.886 | 0.911 | 0.928 | 0.947 | 0.946 | 0.961 | 0.968 | 0.980 | 0.986 | 0.989 |

**Table B.** Probability that the PM has less RMSE than worse agents on the original dataset. The different rows represent differences in RMSE with respect to the best model on the sample size dataset. Each column represents sample sizes: 100, 300 and 600.

|  | **100** | **300** | **600** |
| --- | --- | --- | --- |
| **0.02** | 0.936 | 0.989 | 0.999 |
| **0.04** | 0.993 | 1.000 | 1.000 |
| **0.06** | 0.997 | 1.000 | 1.000 |
| **0.08** | 1.000 | 1.000 | 1.000 |
| **0.10** | 1.000 | 1.000 | 1.000 |
| **0.12** | 1.000 | 1.000 | 1.000 |
| **0.14** | 1.000 | 1.000 | 1.000 |
| **0.16** | 1.000 | 1.000 | 1.000 |
| **0.18** | 1.000 | 1.000 | 1.000 |
| **0.20** | 1.000 | 1.000 | 1.000 |

**Table C.** Probability that the PM has greater AUC than worse agents on the original dataset. The different rows represent differences in AUC with respect to the best model on the sample size dataset. Each column represents sample sizes: 100, 300 and 600.

|  | **100** | **300** | **600** |
| --- | --- | --- | --- |
| **0.01** | 0.781 | 0.740 | 0.881 |
| **0.02** | 0.857 | 0.875 | 0.976 |
| **0.03** | 0.912 | 0.952 | 0.997 |
| **0.04** | 0.945 | 0.980 | 0.999 |
| **0.05** | 0.970 | 0.995 | 1.000 |
| **0.06** | 0.981 | 0.999 | 1.000 |
| **0.07** | 0.990 | 1.000 | 1.000 |
| **0.08** | 0.992 | 1.000 | 1.000 |
| **0.09** | 0.997 | 1.000 | 1.000 |
| **0.10** | 0.998 | 1.000 | 1.000 |
| **0.11** | 0.999 | 1.000 | 1.000 |
| **0.12** | 1.000 | 1.000 | 1.000 |
